# Correction to “Clinical Images: Massive soft‐tissue calcification in primary Sjogren syndrome”

**DOI:** 10.1002/acr2.11608

**Published:** 2023-09-15

Yokochi R, Hagino N. Clinical Images: Massive soft‐tissue calcification in primary Sjogren syndrome. *ACR Open Rheumatol*. 2023 Mar;5(3):114. doi: 10.1002/acr2.11528

Dr. Yokochi's first name was misspelled as Rtisuko in the published version. The correct spelling is Ritsuko.

We apologize for this error.

